# Early outcomes of online adaptive MR-guided SBRT for localized prostate cancer: a prospective study

**DOI:** 10.1016/j.ctro.2026.101240

**Published:** 2026-07-25

**Authors:** Abrahams Ocanto, María González, Macarena Teja, Gloria Guardia, Gloria Sánchez, Daniela Gonsalves, Castalia Fernández, Lisselott Torres, Luis Glaría, Israel Thuissard, Miren Gaztañaga, José García, Sigfredo Elías Romero Zhogbi, Jon Andreescu, Álvaro Flores, Maia Dzhugashvili, David Esteban, Evita Krumina, Loubna Aaki, Jaume Fernández, José González, Antonio Ristori, José Begara, Daniel Rivas, Jasuani Bravo, Ana Vílchez, Luis Clemente, Juan Martínez-Salamanca, Luca Nicosia, Filippo Alongi, Andrew Loblaw, Fernando López-Campos, Felipe Couñago

**Affiliations:** a Department of Radiation Oncology, Hospital Universitario Vithas La Milagrosa, GenesisCare, 28010 Madrid, Spain; b Department of Radiation Oncology, Hospital Universitario San Francisco de Asís, GenesisCare, 28002 Madrid, Spain; c Department of Medicine, Faculty of Medicine, Health and Sports, European University of Madrid, Spain; d Department of Radiation Oncology, Hospital Universitario La Paz, 28046 Madrid, Spain; e Faculty of Sports Sciences, Universidad Europea de Madrid, 16740, Spain; f Department of Radiation Oncology, Hospital Clínico San Carlos, 28040 Madrid, Spain; g Department of Medical Oncology, Hospital Universitario San Francisco de Asís, GenesisCare, 28002 Madrid, Spain; h Department of Radiation Oncology, GenesisCare Talavera de la Reina, Talavera de la Reina, Spain; i Department of Radiation Oncology, San Juan de Dios University Hospital, Genesis Care, Córdoba 14012, Spain; j Department of Radiation Oncology, GenesisCare Campo de Gibraltar, Algeciras 11370, Spain; k Department of Radiation Oncology, GenesisCare Alcázar de San Juan, Ciudad Real 13600, Spain; l Department of Radiation Oncology, GenesisCare Guadalajara, Guadalajara 19004, Spain; m Department of Radiation Oncology, Hospital Vithas Perpetuo Socorro, Alicante 03013, Spain; n Department of Radiation Oncology, Centro 360 de Excelencia Oncológica GCCC, Barcelona 08017, Spain; o Department of Radiation Oncology, GenesisCare Sevilla, Sevilla 41092, Spain; p Department of Radiation Oncology, GenesisCare Jerez de la Frontera, Cádiz 11407, Spain; q Department of Radiation Oncology, GenesisCare Málaga, Málaga 29018, Spain; r Department of Radiation Oncology, Instituto Nacional de Cancerología, Ciudad de México 14080, Spain; s Department of Radiation Oncology, Hospital Meixoeiro de Vigo, Vigo 36214, Spain; t Deparment of Urology, Clínica Uro S.XXI, Madrid 28036, Spain; u Deparment of Urology, Lyx Instituto de Urologia, Madrid 28006, Spain; v Advanced Radiation Oncology Department, IRCCS Ospedale Sacro Cuore Don Calabria, Negrar di Valpolicella, Italy; w University of Brescia, Italy; x Department of Radiation Oncology, Sunnybrook Health Sciences Centre, 2075, Ontario, Canada; y Department of Radiation Oncology, Hospital Universitario Ramón y Canal, 28034 Madrid, Spain

## Abstract

• Online adaptive MR-guided SBRT demonstrated low rates of acute side effects grade ≥ 2 toxicity (3% GU, 0% GI).
• Daily adaptive planning with 2-mm margins enabled favorable tolerability outcomes without hydrogel spacers.
• Patient-reported outcomes showed minimal impact on urinary, bowel, and global quality of life.
• No biochemical failures were observed at 1-year follow-up.
• MRgRT may improve safety profiles beyond margin reduction through adaptive workflows.

## 1 Introduction

Hypofractionated radiotherapy is an established standard of care for localized prostate cancer, supported by robust randomized evidence and by the radiobiological characteristics of prostate adenocarcinoma, particularly its low estimated α/β ratio, which suggests enhanced sensitivity to larger fraction sizes [1]. Within this framework, ultrahypofractionated stereotactic body radiotherapy (SBRT) has demonstrated excellent biochemical control and favorable tolerability across prospective studies and randomized trials [2], [3].

Despite these favorable outcomes, prostate SBRT remains technically demanding. Inter- and intrafraction prostate motion, day-to-day anatomical variation, and the close proximity of dose-limiting organs at risk (OARs), particularly the rectum, bladder, and urethra, may compromise target coverage or increase treatment-related side effects when highly conformal dose distributions are delivered [4], [5], [6].

Magnetic resonance-guided radiotherapy (MRIgRT) has emerged as a promising strategy to address these challenges. Compared with conventional image guidance, MRIgRT provides superior soft-tissue visualization, real-time motion monitoring, intrafraction gating, and the opportunity for daily online adaptive replanning, potentially enabling tighter planning target volume (PTV) margins and improved sparing of adjacent normal tissues [5], [7], [8].

The phase III MIRAGE trial by Kishan et al. [9] demonstrated significantly lower rates of acute grade ≥ 2 gastrointestinal (GI) and genitourinary (GU) adverse events with MR-guided SBRT compared with CT-guided SBRT. This benefit was largely attributed to reduced planning target volume margins (2 mm vs. 4 mm), enabled by improved visualization and motion management. More recently, 2-year outcomes suggested that these advantages may be sustained over time and may also contribute to better preservation of sexual function [10].

However, whether fully online adaptive MR-guided workflows provide additional clinical benefit beyond improved visualization and margin reduction remains uncertain. Prospective data evaluating daily online adaptive MR-guided SBRT in routine clinical practice remain limited, particularly regarding early adverse events and patient-reported outcomes.

Therefore, we conducted a prospective study to evaluate the feasibility, early safety profile, patient-reported outcomes, and short-term biochemical response of online adaptive MR-guided SBRT for localized prostate cancer.

## 2 Methods

### 2.1 Study design and patients

This prospective, single-center, non-randomized study included consecutive men aged ≥18 years with histologically confirmed non-metastatic prostate adenocarcinoma treated between July 1, 2023 and July 1, 2025. Eligible patients had predominantly localized disease across all risk groups (low-, intermediate-, high-, and very high-risk) and were considered suitable for stereotactic body radiotherapy (SBRT). One patient had clinically node-positive pelvic disease without distant metastases. Key exclusion criteria included MRI-incompatible implanted devices and severe claustrophobia precluding magnetic resonance imaging. The study was approved by the Institutional Review Board of Hospital Universitario de La Princesa (CEIm; Registry No. 5289), and all patients provided written informed consent prior to enrolment.

### 2.2 Simulation and treatment workflow

All patients were treated with magnetic resonance-guided radiotherapy (MRIgRT) using a 0.35-T MR-Linac system (MRIdian Linac, ViewRay, Mountain View, CA, USA). Specifics of the technical aspects fo the system are reported in previous report [11]**.** Simulation consisted of a planning computed tomography (CT) scan and a free-breathing MRI acquired using a true fast imaging with steady-state precession (TRUFI) sequence. Diagnostic multiparametric MRI (1.5–3 T) was co-registered when available to support target delineation. Patients were instructed to attend treatment with a comfortably full bladder and an empty rectum. Fiducial markers and rectal spacers were not routinely used. Urinary catheterization was performed only when clinically indicated.

### 2.3 Target delineation and dose prescription

The clinical target volume (CTV) consisted of the prostate gland alone or the prostate plus seminal vesicles according to disease risk group and physician discretion. Pelvic nodal target volumes were contoured only when clinically indicated (high risk). A uniform 2-mm isotropic expansion was applied to generate the planning target volume (PTV). Prescribed dose was 36.25 Gy in five fractions for low-risk disease, 36.25–40 Gy in five fractions for intermediate-risk disease (according to medical criteria), and 40 Gy in five fractions for high- and very high-risk disease. The urethral-sparing was used in patients to 40Gy. Dose selection within the intermediate-risk group was individualized according to disease characteristics and treatment planning considerations. At least 95% of the PTV received the prescribed dose while respecting institutional OAR constraints [9]. Elective pelvic nodal irradiation up to 25 Gy in five fractions was permitted in selected patients (positives nodes and high risk), with a simultaneous integrated boost to radiologically involved pelvic lymph nodes up to 35 Gy in five fractions, a isotropic 4 mm expansion was applied to generate the PTV. Androgen deprivation therapy (ADT) was administered at the discretion of the treating physician according to disease risk group and institutional practice.

### 2.4 Online adaptive delivery

Treatment was delivered using step-and-shoot intensity-modulated radiotherapy (IMRT), with fractions delivered on alternate days [11]. Prior to each fraction, a new setup MRI was acquired and daily online adaptive replanning was performed based on the anatomy of the day. During treatment delivery, continuous cine MRI was acquired at four frames per second in sagittal and coronal planes for real-time motion monitoring. If >5% of the prostate volume moved beyond a 2 mm gating boundary, beam delivery was automatically interrupted until target position returned within the predefined threshold.

### 2.5 Endpoints and adverse events assessment

The primary endpoint was the incidence of grade ≥ 2 genitourinary (GU) and gastrointestinal (GI) adverse events, scored according to the Common Terminology Criteria for Adverse Events version 4.03 (CTCAE v4.03). Acute adverse events were defined as events occurring from treatment initiation to 90 days after SBRT completion. Events recorded at 6 months were analysed separately as early follow-up outcomes.

Secondary endpoints included changes in patient-reported outcomes assessed using the International Prostate Symptom Score (IPSS) [12], Expanded Prostate Cancer Index Composite-26 (EPIC-26) [13], and European Organisation for Research and Treatment of Cancer Quality of Life Questionnaire Core 30 (EORTC QLQ-C30) [14].

For EPIC-26, clinically meaningful deterioration was defined according to established minimally important difference thresholds: >18 points for urinary incontinence, >14 points for urinary irritative/obstructive symptoms, >12 points for bowel function, and > 24 points for sexual function [15]. For IPSS, an increase of ≥8 points from baseline was considered clinically relevant. QLQ-C30 scoring followed EORTC recommendations. Patients underwent physician assessment and completed questionnaires at baseline and during scheduled follow-up visits, including 3 and 6 months after treatment.

### 2.6 Statistical analysis

Descriptive statistics were used to summarize baseline, treatment, and outcome variables. Continuous variables are reported as median and interquartile range (IQR) or mean ± standard deviation, according to distribution. Categorical variables are presented as frequencies and percentages.

Comparisons between subgroups were performed using the chi-square test or Fisher's exact test for categorical variables, and Student's *t*-test or Mann–Whitney *U* test for continuous variables, as appropriate. Logistic regression analysis was used to identify factors associated with grade ≥ 2 acute GU adverse events. Variables with p<0.10 in univariable analyses or considered clinically relevant were entered into multivariable models. Odds ratios (ORs) with 95% confidence intervals (CIs) were reported. All statistical analyses were performed using SPSS version 27.0 (IBM Corp., Armonk, NY, USA). Two-sided *p* values <0.05 were considered statistically significant.

## 3 Results

### 3.1 Baseline patient and treatment characteristics

Between July 2023 and July 2025, 108 patients were enrolled and treated with online adaptive MR-guided SBRT. Median age was 74 years (IQR 68–81). According to ECOG performance status, 82 patients (76%) had ECOG 0 and 26 (24%) had ECOG 1. Most patients had intermediate-risk disease (62%), while 15% had high- or very high-risk disease. One patient (1%) had clinically node-positive pelvic disease. Androgen deprivation therapy (ADT) was administered in 38 patients (35%). Median prostate volume was 45.3 cc. Median baseline PSA was 8.56 ng/mL, decreasing to 1.37 ng/mL at 3 months, 1.03 ng/mL at 6 months, and 0.76 ng/mL at 12 months. No biochemical failures were observed during 12 months of follow-up. Baseline and treatment characteristics are summarized in Table 1.

**Table 1:** Baseline characteristics.

**Baseline Patient Characteristics (*n* = 108)**	
**Age (years), mediana [Q1-Q3]**	74 [68–81]
ECOG	
**0**	82 (76%)
**1**	26 (24%)
Risk group	
**Low risk**	26 (24%)
**Favorable intermediate**	36 (33%)
**Unfavorable intermediate**	31 (29%)
**High risk**	15 (14%)
Stage	
**T1c**	23 (21%)
**T2a**	25 (23%)
**T2b**	20 (19%)
**T2c**	23 (21%)
**T3a**	14 (13%)
**T3b**	2 (2%)
**T4**	1 (1%)
**N0**	107 (99%)
**N1**	1 (1%)
**Use of ADT**	38 (35%)
**6 months**	25 (23%)
**24 months**	13 (8%)
**Volume prostate (median)**	45,27 cc
**<50 cc**	23 (21%)
**>50 cc**	26 (24%)
**PSA at diagnosis (ng/mL) mediana [Q1-Q3]**	8.56
**PSA at three months (ng/mL) mediana [Q1-Q3]**	1.37
**PSA at six months (ng/mL) mediana [Q1-Q3]**	1.03
**PSA at twelve months (ng/mL) mediana [Q1-Q3]**	0.76

### 3.2 Physician-reported adverse events

**Fig. 1:**
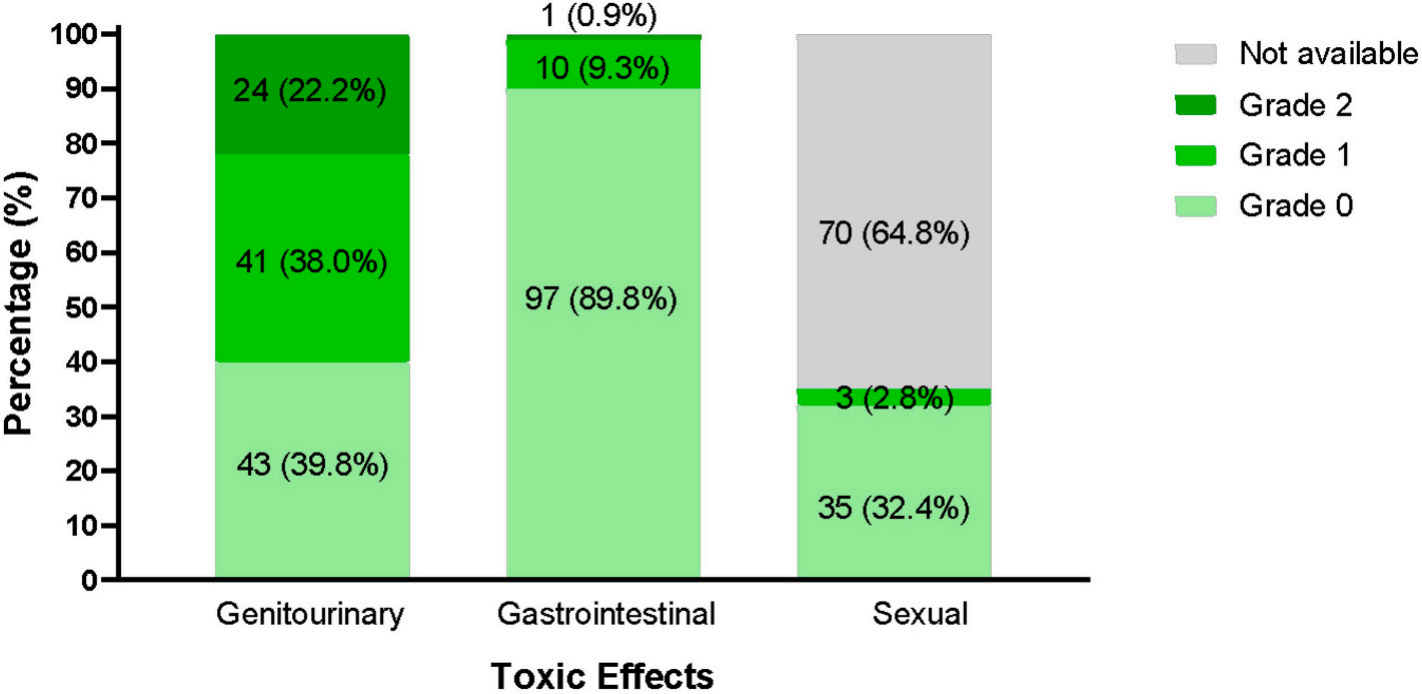
Any adverse events during the 12 months after finished radiotherapy.

At 2 weeks after treatment completion, grade ≥ 2 genitourinary (GU) adverse events were observed in 22% of patients, whereas grade ≥ 2 gastrointestinal (GI) adverse events occurred in 1%. At 3 months, grade ≥ 2 GU adverse events declined to 3%, with no grade ≥ 2 GI events. Rates remained low at 6 months, with grade ≥ 2 GU adverse events reported in 3% and no grade ≥ 2 GI events. No grade 3 GI adverse events were recorded. One grade 3 GU event (urinary retention) was observed at 12 months. Any-grade GU, GI, and sexual adverse events were reported in 32%, 2%, and 8% of evaluable patients at 3 months, decreasing to 9%, 1%, and 1% at 6 months, respectively. Overall, adverse events were predominantly mild, transient, and improved over time **(**Fig. 1**)**. Detailed physician-reported adverse event data are presented in Table 2.

**TABLE 2:** Adverse events registered at 3, 6 and 12 months.

	**3 months**			**6 months**			**12 months**		
	**Grade 1**	**Grade 2**	**Grade 3**	**Grade 1**	**Grade 2**	**Grade 3**	**Grade 1**	**Grade 2**	**Grade 3**
**Genitourinary**									
Any	0%	3 (3%)	0%	0%	3 (5%)	0%	0%	5 (13%)	0%
Cystitis	11 (11%)	2 (2%)	0%	3 (5%)	1 (2%)	0%	1 (3%)	4 (10%)	0%
Hematury	0%	0%	0%	1 (2%)	0%	0%	2 (5%)	1 (3%)	0%
Urinary Incontinence	3 (3%)	0%	0%	1 (2%)	0%	0%	1 (3%)	0%	0%
Urinary Retention	10 (11%)	1 (1%)	0%	4 (6%)	1 (2%)	0%	1 (3%)	2 (5%)	1 (3%)
Dysuria	3 (3%)	0%	0%	2 (3%)	1 (2%)	0%	0%	0%	0%

Sexual									
Erectile Dysfunction	2 (7%)	0%	0%	2 (7%)	0%	0%	1 (3%)	0%	0%

Gastrointestinal									
Any	2 (2%)	0%	0%	2 (3%)	0%	0%	0%	0%	0%
Diarrhea	0%	0%	0%	0%	0%	0%	0%	0%	0%
GI hemorrhage	1 (1%)	0%	0%	0%	0%	0%	0%	0%	0%
Rectal pain	1 (1%)	0%	0%	2 (3%)	0%	0%	0%	0%	0%

### 3.3 Factors associated with adverse events

In univariate analysis, higher IPSS score is reported at baseline were associated with an increased risk of grade ≥ 2 acute GU adverse events (OR 1.108; 95% CI 1.009–1.216; *p* = 0.031). A similar trend was observed in multivariable analysis **(**Table 3**)**. Patients with prostate volume > 50 cc had significantly larger planning target volumes (*p* < 0.001), although prostate volume was not independently associated with grade ≥ 2 GU adverse events. No correlation with dose level.

**TABLE 3:** Univariate and multivariate analysis of factors associated with grade ≥ 2 GU adverse events.

	**Univariate logistic regression**	**Multivariate logistic regression**⁎
**GU adverse event ≥ grade 2**	**OR [95%CI], p**	**OR [95%CI], p**⁎
Age, years	0.991 (0.947–1.038),0.0,71	
ECOG	1.409 (0.509–3.898), 0.50	
Gleason score		
3 + 4	1.400 (0.408–4.804), 0.59	
4 + 3	1.008 (0.269–3.777), 0.99	
4 + 4	1.575 (0.303–8.175), 0.58	
4 + 5	1.400 (0.119–16.459), 0.78	
ADT	4.500 (0.977–20.724), 0.05	

Clinical TNM		
T2a	0.592 (0.169–2.080), 0.41	
T2b	0.208 (0.038–1.134), 0.07	
T2c	0.221 (0.040–1.205), 0.08	
T3a	0.833 (0.194–3.578), 0.80	
T3b	1.875 (0.103–34.131), 0.67	
T4	0 (0–0) 1	
N1	0 (0–0) 1	

Prostate volume		
>50 cc	0.782 (0.220–2.779), 0.70	
IPSS	**1.108 (1.009–1.216), 0.031**	**1.09 (1.01–1.18), 0.02**
QLQ-C30	0.636 (0.223–1.817), 0.39	
EPIC-26	0.861 (0.306–2.427), 0.77	
PSA at diagnosis	0.947 (0.855–1.048), 0.29	

⁎ Adjusted by baseline factors with *p* < 0.05.

### 3.4 Patient-reported outcomes

**Fig. 2:**
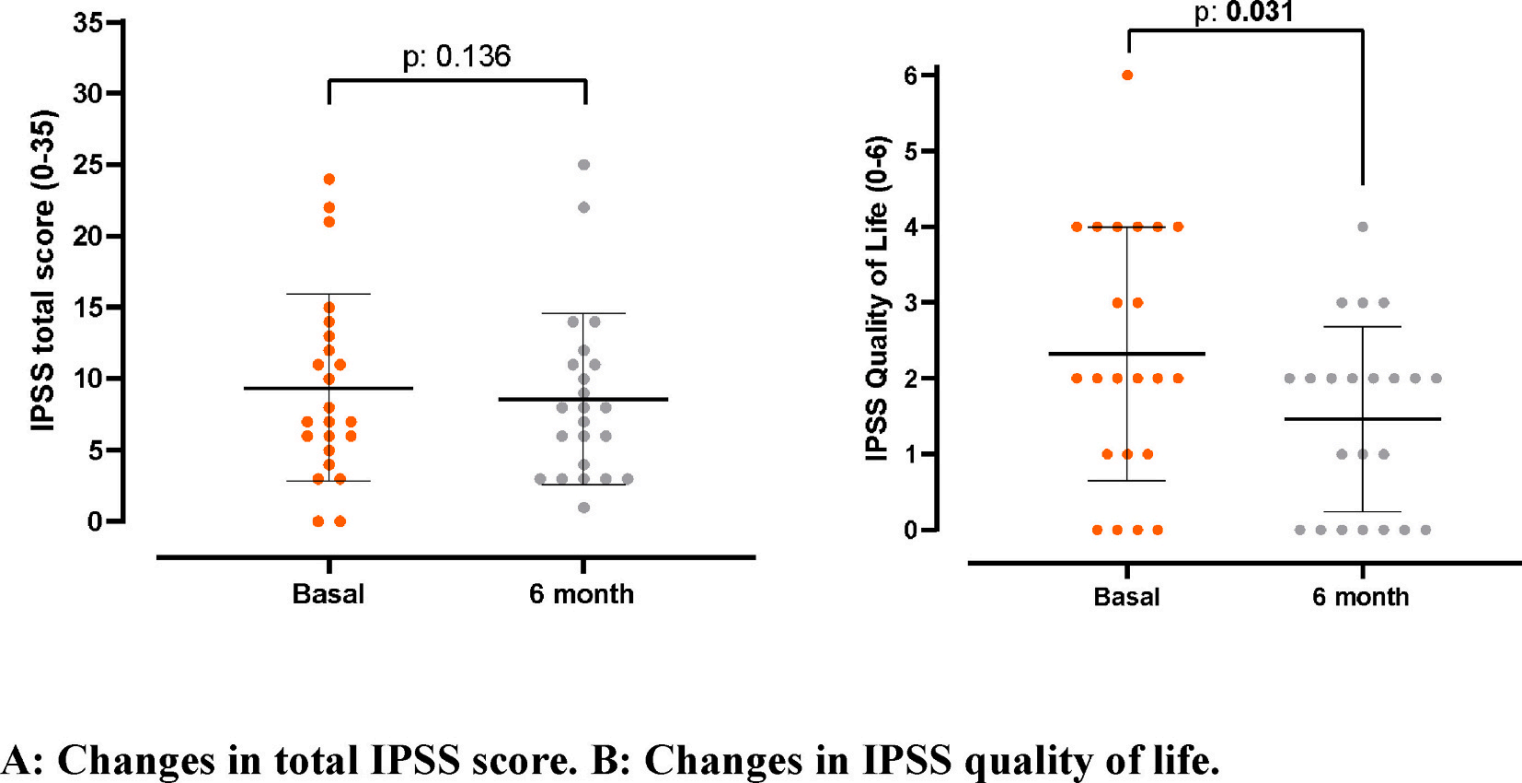
Evolution of IPSS score before radiotherapy: basal & six months. A: Changes in total IPSS score. B: Changes in IPSS quality of life.

**Fig. 3:**
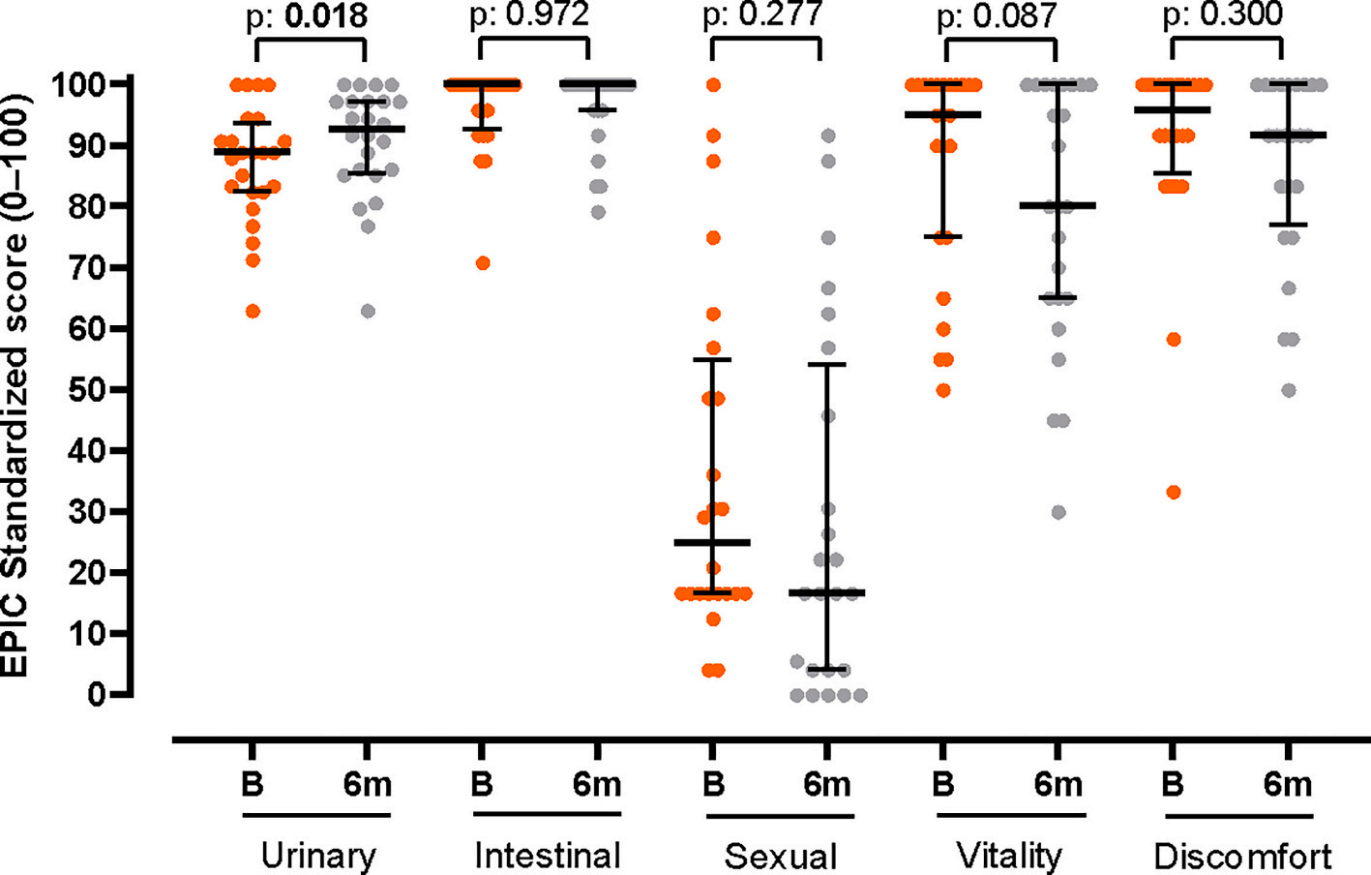
Changes in EPIC-26 score: before and six months after finished radiotherapy.

**Fig. 4:**
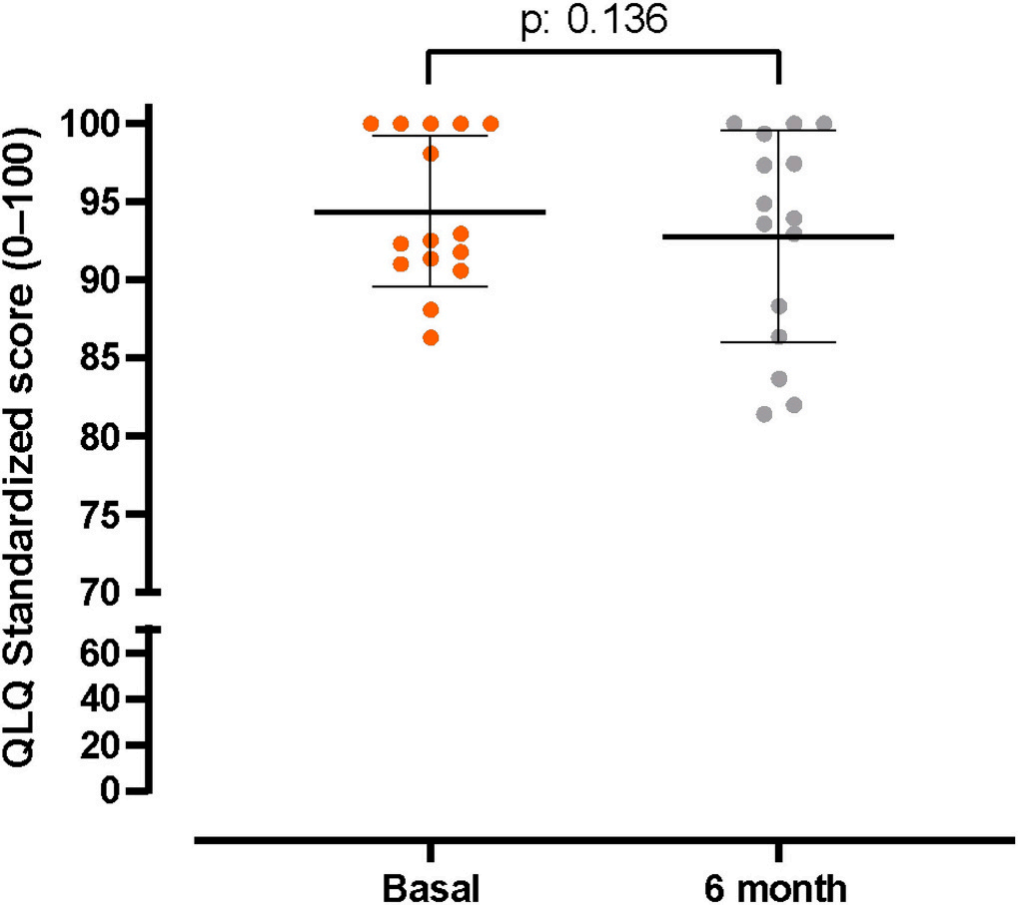
EORTC QLQ-C30 before and six months after finished radiotherapy.

Longitudinal changes in urinary and bowel patient-reported outcomes are shown in Fig. 2. Among evaluable patients, IPSS score was a median value of 7 (3–24 points) remained stable in 61%, while 30% reported minor changes and 9% experienced clinically relevant worsening at 6 months (*p* = 0.514). At 6 months, EPIC-26 urinary incontinence scores remained stable in 79% of patients, whereas 21% reported mild worsening (*p* = 0.018). For urinary irritative/obstructive symptoms, 58% remained stable, 21% reported mild worsening, and 21% reported clinically relevant deterioration (*p* = 0.300). In the bowel domain, 75% remained stable, 12.5% reported mild worsening, and 12.5% experienced clinically relevant deterioration (*p* = 0.972). In the sexual domain, 42% remained stable, whereas the remainder reported mild or clinically relevant worsening (*p* = 0.277) **(**Fig. 3**)**. Global quality of life assessed with EORTC QLQ-C30 remained overall stable at 6 months, with 53% of patients reporting no relevant change and 47% reporting mild changes (*p* = 0.136) **(**Fig. 4**)**. In summary no significant difference was found between baseline and 6-month evaluation.

## 4 Discussion

In this prospective single-center study, online adaptive MR-guided SBRT for prostate cancer was feasible and associated with a favorable early safety profile, low rates of clinically significant physician-reported adverse events, and limited deterioration in patient-reported quality of life. Grade ≥ 2 GU adverse events were mainly transient, declining from 22% at 2 weeks to 3% at 3 and 6 months, while grade ≥ 2 GI adverse events were uncommon throughout follow-up. In parallel, urinary, bowel, and global quality-of-life outcomes were largely preserved.

SBRT is an established treatment option for localized prostate cancer, supported by randomized and long-term prospective data demonstrating oncological efficacy comparable to conventional or moderately hypofractionated radiotherapy, with the advantages of shorter treatment duration and improved convenience [2], [3], [13], [14], [15], [16], [17], [18], [19]. However, delivery of high-dose fractions requires accurate target localization and robust motion management, given the susceptibility of the prostate to inter- and intrafraction motion and the close proximity of critical pelvic organs.

MRIgRT offers several technical advantages over conventional image-guided approaches, including superior soft-tissue visualization, real-time cine monitoring, intrafraction gating, and the possibility of daily online adaptation [5], [6], [20], [21]. These features may facilitate tighter treatment margins and improved organ-at-risk sparing, which are particularly relevant in the setting of prostate SBRT.

The phase III MIRAGE trial established the clinical relevance of MR-guidance by demonstrating significantly lower rates of acute grade ≥ 2 GU and GI adverse events with MR-guided versus CT-guided SBRT [7]. Updated 2-year outcomes suggested persistence of these benefits and improved preservation of sexual function [8]. Importantly, MIRAGE did not incorporate daily online adaptive replanning. In the present study, all patients were treated with a fully adaptive workflow using 2-mm margins, and rectal spacers were not routinely used. Despite this, clinically significant GI adverse events remained uncommon throughout follow-up. Although cross-study comparisons should be interpreted cautiously, these findings suggest that online adaptation may help optimize rectal sparing and could potentially reduce reliance on adjunctive rectal displacement strategies in selected patients. The role of rectal spacers within adaptive MR-guided workflows warrants prospective evaluation.

Our results are also consistent with previous prospective MR-guided prostate SBRT series. Alongi et al. [20] reported favorable feasibility and patient-reported outcomes using daily adaptive MR-guided SBRT, while Bruynzeel et al. [21] observed low rates of clinically significant acute adverse events in a phase II study. More recently, systematic evidence has suggested that adaptive MR-guided workflows may reduce acute morbidity compared with non-adaptive approaches [5]. The present study adds prospective real-world data from routine clinical practice using a contemporary online adaptive platform.

An additional relevant finding was the limited impact on patient-reported outcomes. Most patients maintained stable urinary symptom burden and global quality of life, while clinically meaningful deterioration in bowel or urinary domains was infrequent. Sexual outcomes should be interpreted cautiously, as 35% of patients received androgen deprivation therapy, which may independently impair sexual function [22].

This study has limitations. First, the single-center, non-randomized design limits external validity and precludes causal comparisons with other image-guidance strategies. Second, follow-up remains relatively short for a disease with long natural history, and late urinary or rectal morbidity cannot yet be fully assessed. Third, completion rates for patient-reported outcome questionnaires decreased over time, which may introduce response bias. Finally, treatment allocation and dose selection were individualized according to routine practice, introducing some heterogeneity within the cohort.

Despite these limitations, the prospective design, systematic collection of physician- reported adverse events, and inclusion of validated patient-reported outcomes strengthen the clinical relevance of these findings.

## 5 Conclusion

Online adaptive MR-guided SBRT demonstrated a favorable early safety profile with preservation of patient-reported quality of life in patients with localized prostate cancer. Longer follow-up and comparative studies are warranted to clarify the incremental value of daily online adaptation on long-term morbidity, quality of life, and oncological outcomes.

## CRediT authorship contribution statement

**Abrahams Ocanto:** Writing – review & editing, Writing – original draft, Validation, Methodology, Investigation, Formal analysis, Data curation, Conceptualization. **María González:** Visualization, Validation. **Macarena Teja:** Validation, Supervision. **Gloria Guardia:** Validation, Supervision. **Gloria Sánchez:** Validation, Supervision. **Daniela Gonsalves:** Visualization, Validation. **Castalia Fernández:** Visualization, Validation. **Lisselott Torres:** Validation, Supervision. **Luis Glaría:** Visualization, Validation. **Israel Thuissard:** Methodology, Formal analysis. **Miren Gaztañaga:** Validation, Supervision. **José García:** Visualization, Validation. **Sigfredo Elías Romero Zhogbi:** Visualization, Validation, Supervision. **Jon Andreescu:** Visualization, Validation. **Álvaro Flores:** Visualization, Validation. **Maia Dzhugashvili:** Visualization, Validation. **David Esteban:** Visualization, Validation. **Evita Krumina:** Validation, Supervision. **Loubna Aaki:** Visualization, Validation. **Jaume Fernández:** Visualization, Validation. **José González:** Visualization, Validation. **Antonio Ristori:** Visualization, Validation, Investigation. **José Begara:** Validation, Supervision. **Daniel Rivas:** Visualization, Validation. **Jasuani Bravo:** Visualization, Validation. **Ana Vílchez:** Methodology, Investigation. **Luis Clemente:** Visualization, Validation. **Juan Martínez-Salamanca:** Visualization, Validation. **Luca Nicosia:** Validation, Supervision. **Filippo Alongi:** Validation, Supervision. **Andrew Loblaw:** Visualization, Validation. **Fernando López-Campos:** Visualization, Validation. **Felipe Couñago:** Validation, Methodology, Investigation, Formal analysis, Conceptualization.

## Declaration of competing interest

The authors declare that they have no known competing financial interests or personal relationships that could have appeared to influence the work reported in this paper.
